# Toward the Non-Targeted Detection of Adulterated Virgin Olive Oil with Edible Oils via FTIR Spectroscopy & Chemometrics: Research Methodology Trends, Gaps and Future Perspectives

**DOI:** 10.3390/molecules28010337

**Published:** 2023-01-01

**Authors:** Stella A. Ordoudi, Lorenzo Strani, Marina Cocchi

**Affiliations:** 1 Laboratory of Food Chemistry and Technology, School of Chemistry, Aristotle University of Thessaloniki (AUTh), GR-54124 Thessaloniki, Greece; 2 Department of Chemical and Geological Sciences, University of Modena and Reggio Emilia (UNIMORE), Via Campi 103, 41125 Modena, Italy

**Keywords:** virgin olive oil, non-destructive FTIR spectroscopy, adulteration, non-targeted authenticity testing, edible oils, chemometrics

## Abstract

Fourier-Transform mid-infrared (FTIR) spectroscopy offers a strong candidate screening tool for rapid, non-destructive and early detection of unauthorized virgin olive oil blends with other edible oils. Potential applications to the official anti-fraud control are supported by dozens of research articles with a “proof-of-concept” study approach through different chemometric workflows for comprehensive spectral analysis. It may also assist non-targeted authenticity testing, an emerging goal for modern food fraud inspection systems. Hence, FTIR-based methods need to be standardized and validated to be accepted by the olive industry and official regulators. Thus far, several literature reviews evaluated the competence of FTIR standalone or compared with other vibrational techniques only in view of the chemometric methodology, regardless of the inherent characteristics of the product spectra or the application scope. Regarding authenticity testing, every step of the methodology workflow, and not only the post-acquisition steps, need thorough validation. In this context, the present review investigates the progress in the research methodology on FTIR-based detection of virgin olive oil adulteration over a period of more than 25 years with the aim to capture the trends, identify gaps or misuses in the existing literature and highlight intriguing topics for future studies. An extensive search in Scopus, Web of Science and Google Scholar, combined with bibliometric analysis, helped to extract qualitative and quantitative information from publication sources. Our findings verified that intercomparison of literature results is often impossible; sampling design, FTIR spectral acquisition and performance evaluation are critical methodological issues that need more specific guidance and criteria for application to product authenticity testing.

## 1. Introduction

Today, a large number of analytical tests are planned to ensure the quality and authenticity of virgin olive oil products throughout their production and global supply chain, based on a set of organoleptic, physical and chemical characteristics. Standard quality and purity specifications for labeling “extra-virgin” and “virgin” olive oil (EVOO/VOO) are proposed by the International Olive Council (IOC), adapted in the European legislation (EC No 2568/1991 Regulation and amendments) [1,2] and accepted in worldwide commercial transactions (Codex Committee for fats and oils). Within the European Union (EU), screening tests that involve sensory analyses by accredited tasting panels and/or basic laboratory analyses (e.g., acidity, viscosity, oxidative stability, triacylglycerol content, peroxide, anisidine and iodine values, moisture content, and specific gravity) are performed regularly at the production/supply chain in order to check for non-conformities and reduce the prevalence of common law violations. The latter refer primarily to marketing of EVOO or VOO blends with cheap vegetable and seed oils or low-grade olive oil as pure virgin one. Although the conformity checks in this sector are considered sufficient, there is a continuous global debate about the effectiveness of the test methods, especially in providing early warning signals; as the fraudulent practices become more “scientific” or sophisticated, these tests often fail to detect risks from unexpected, undisclosed adulterants. Moreover, as the assessment protocols are invasive in nature and time-consuming, the average duration of conformity checks until completion is prolonged, having also negative impact on the overall cost of the quality control [3]. In the last years, it has been suggested that regulations need to be amended and to integrate traditional with modern analytical approaches following technological innovations [4].

### 1.1. Non-Targeted Authenticity Testing

Non-targeted screening methods based on spectroscopic techniques (mainly vibrational and Nuclear Magnetic Resonance (NMR) spectroscopy) [5,6] have recently attracted the interest of researchers in the field of food authentication, as they could offer alternative means for rapid quality control and authenticity testing. Such methods utilize the whole spectrum as sample fingerprint, similar to the concept of non-targeted metabolomics, to uncover undisputed cases of fraud, e.g., mislabeling of provenance and product category or adulteration. In this frame, non-destructive and non-invasive testing approaches based on Near- and Mid-Infrared or Raman spectroscopies that have been used for many decades in the industrial process control of several commodities (e.g., beer, dairy and cereal products), but also hyperspectral imaging, could aid the on-field/official control of EVOO/VOO. Thus far, there is a wealth of scientific evidence that such techniques can be powerful in identifying cheaper seed oils or deodorized olive oils in EVOO [7,8]. Given that the vibrational spectrum contains multivariate information about several quality parameters and is obtained out of minute amounts of sample, spectroscopic sensors can be used to collect data from multiple monitoring points along the production and supply chain as parts of modern, digital food quality systems [9]. Modern data science tools that employ signal processing, data compression and multivariate calibration models are indispensable to extract information from the complex spectra and turn it into operational knowledge in the context of chemometrics [10]. Chemometric assessment can be greatly advantageous for quantitative or qualitative analyses of data. Nevertheless, it represents a vigorous task that requires multidisciplinary skills and expertise, along with standard guidelines. Skibsted and Engelsen pointed out that “the challenge and workload of robust calibration remains an effective stopper for widespread use of spectroscopic devices” [11]. Robustness is needed to handle data variability originating from various sources, i.e., the types of reference samples that are used for model training and validation, as well as instrumental instability and instrument-to-instrument differences.

In a forensic view, the ultimate goal is to define the probability of detecting adulterated virgin olive oil as atypical and pure one as typical of its origin and category. To this aim class-modeling coupled with a non-targeted analytical approach seems to be the most promising strategy [12]. The “proof of evidence” could be evaluated considering the predictive performance of the trained model against samples of unknown origin, or directly their distance to the model of the authentic category [13]. However, there is not yet a standard approach or guideline protocol, thus the significance of the results usually varies depending on the statistical algorithm originally selected or the validity of the evaluation criteria [14]. Nowadays, many experts in the field stress that when class models are trained for the prediction of abnormalities in spectra, it is essential to define standard performance criteria, regardless of the statistical approach. For example, sensitivity against atypical samples and specificity rates at a desired confidence level should match the nominal values, and the chosen confidence should be in agreement with the regulation requirements. In fact, it is necessary to avoid possible safety hazards and/or to reduce the number of suspect samples that need to be re-tested. Thus, the selection of a model validation procedure is crucial for the future applicability of the method in the context of non-targeted authenticity testing. Currently, there is a consensus that standard guidelines are needed in every step of the workflow, including the very first one, e.g., creation of reference data sets. Scenario analyses, theory of sampling and experimental design principles should be employed much more to ensure representativeness, sufficient size, etc. [15,16,17]. Above all, the reference samples that are used to calibrate the predictive model should be “relative to the universe of authentic samples, the variability of the method itself relative to the specification range, and the natural variability inherent in the universe of the authentic ingredient” [18]. Representative sampling is a key requirement for stepping forward to spectroscopic data analysis through general workflows, as those proposed by Nunes [19] or Callao and Ruisanchez [20], and building internationally standardized protocols.

### 1.2. FTIR Spectroscopy in the Quality Control of EVOO/VOO

**Figure 1:**
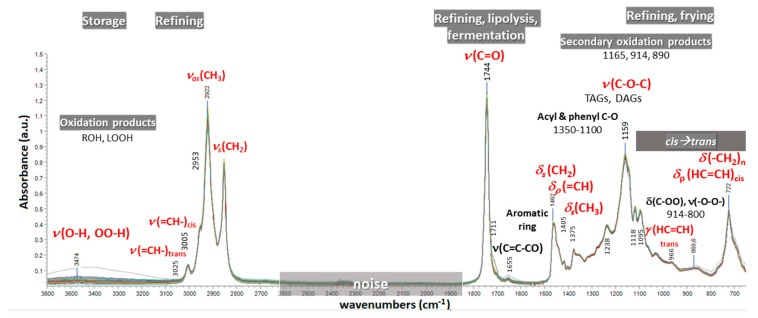
Attenuated Total Reflectance (ATR)-FTIR (3600–650 cm^−1^) spectral features that are commonly accepted as diagnostic of virgin olive oil quality deterioration throughout its production and storage [21,23,24,25]. Peak assignments are highlighted in red. *ν*, stretching vibration; *δ*, bending, in plane deformation vibration; *γ*, bending, out of plane deformation vibration; *ρ*, rocking vibration; *s*, symmetric vibration; *as*, asymmetric vibration; ROH, aliphatic alcohols; LOOH, lipid hydroperoxides; TAGs, triacylglycerols; DAGs, diacylglycerols.

In the case of edible oils, chemometric models based on mid-infrared (MIR) spectroscopic data often perform better than near-infrared (NIR) and Raman ones, when studying quality issues in general [19]. FTIR spectroscopy represents a powerful, versatile, non-destructive technique of low analysis cost, an alternative to wet-chemical and time-consuming techniques. It monitors the fundamental vibrational and rotational movements of molecules upon absorption of light in the mid-infrared region of the electromagnetic spectrum (4000 to 400 cm^−1^). This region retains information about the structure and conformation of proteins, polysaccharides and lipids that can be useful for diagnostic purposes. Multiple signals of varying frequency/intensity, due to the contribution of multiple analytes or even the sample matrix itself, compose the FTIR spectrum of an oil sample at a particular time slot. Therefore, by spectral data analysis, it is possible to identify, monitor and track undesired structural changes in lipid molecules in terms of, e.g., the chain length, unsaturation, *trans*/*cis* configuration of double bonds or ester linkages of fatty acids. Many factors related with intrinsic characteristics of the olive fruits (genetic, physiological) and environmental conditions (oxygen, light, temperature) may induce molecular transformations during the production, handling and storage of the EVOO/VOO products, giving rise to subtle variation in their spectra. Figure 1 displays characteristic peaks and bands in the raw FTIR spectra of virgin olive oils, along with assignments [21] that are considered diagnostic of quality deterioration during various conditions of processing and storage. As an example, variance in the band at 966 cm^−1^ due to isolated (non-conjugated) *trans* double bonds [22], but also in the region between 914 to 800 cm^−1^ [23,24], may reveal the presence of thermal oxidation products. In contrast, variance at around 1746 cm^−1^ due to carboxylic ester groups is expected to carry information about improper conditions of olive storage and elevation of fatty acid ethyl esters (FAEEs) content [25]. Chemometric analysis of the spectral data is indispensable and may offer insight to the “natural” variability in pure virgin olive oil composition. Beyond inherent variation, the presence of extraneous matter, e.g., oils of foreign botanical origin or extraneous pigments, can also induce invisible differential changes that can be exposed, depending on the quality of the attained spectroscopic data and the overall strategy of chemometric modeling and validation [26]. The signals in the fingerprint region from 1500 to 700 cm^−1^, as well as the C–H stretching bands from 3100 to 2800 cm^−1^, are often under scrutiny, as they can be diagnostic of the target adulterant. Usually, shifted bands between 3003 and 3020 cm^−1^ that are due to C-H stretching in *cis*-olefins denote a high level of unsaturated fatty acids and adulteration with seed oils. In practice, as spectral variance arises synchronously from C–H, C–O and C=C vibrations in different structural units of the predominant acylglycerols, it is impossible to identify the extraneous botanical origin in complex olive oil blends [27]. Chemoinformatic databases with FTIR spectroscopic data for EVOO/VOO or other types of oil samples are necessary for fingerprint analyses [26,28]. However, given the lack of standard assessment protocols, confusion about the terminology and misuses, misinterpretation of the research findings are often encountered in the scientific literature [6,29,30,31].

## 2. Concept and Methodology

The current review aims to summarize the results and evaluate the methodological concepts and workflows of literature studies where FTIR spectroscopy was proposed as a non-destructive tool to detect virgin olive oil adulteration with blends of lower quality or cheaper vegetable oils. The main objective is to highlight critical issues, starting from the design of experiments and selection of samples, instrumental settings and acquisition of data, pre-processing methods and data exploration up to the multivariate calibration/classification methods and validation results. For this purpose, a systematic literature search and meta-analysis of the data published thus far were carried out. To the best of our knowledge, no similar research has been conducted to evaluate trends in the research methodology, identify gaps and suggest future perspectives for implementation of FTIR spectroscopy to the anti-fraud control of high value olive oil products.

Extensive literature search was carried out in the Web of Science (core collection), Scopus and Google Scholar databases (last searched on July 2022). Various combinations of the keywords ‘virgin olive oil’ AND ‘adulteration’ AND ‘FTIR’ in the search fields “title” and “abstract” were used with no publication time filters. The retrieved papers were screened for compliance with our search criteria to remove studies of low relevance. In total, 47 original research articles and 12 reviews were retrieved. The data sets from Web of Science and Scopus that included information about the type of paper, authors’ name and title, authors’ affiliations, journal name, doi, publication year, keywords and abstract were exported using the Research Information Systems (RIS) format and further processed via the open source BibExcel toolbox [32]. This tool allowed all the metadata to be extracted in a file format that could be imported to Microsoft Excel and uniquely tagged to the respective publication. Next, we defined some additional criteria to find those original research articles that report experimentation with mixtures of virgin olive oil and other types of edible oils using rapid, non-destructive measurement protocols. Information was extracted from the (i) aim of the study, (ii) materials and methods, as well as (iii) the results and discussion sections, and it involved the following six subject categories: (i) field of application i.e., the botanical origin or process type of adulterant(s) under study, (ii) sampling methodology, focusing on the design and composition of test mixtures (binary, ternary, complex and concentration ranges) and summarizing details about the number of reference samples and available metadata, (iii) spectra acquisition conditions (type of instrument, type of cell, instrument parameters), (iv) spectral pre-processing schemes and (v) modeling strategy (e.g., types of calibration models and validation schemes). Bibliometric results are presented and discussed in separate sections per category. In-depth explanation of various mathematical treatment and chemometric methods that are mentioned throughout the article is not given here, but the reader is referred to the basic bibliography for more details, e.g., [33,34,35,36,37,38,39,40,41,42,43,44,45,46]. A glossary of some terms and basic descriptions is also given as supplementary information (see Supplementary Materials).

## 3. Results and Discussion

**Figure 2:**
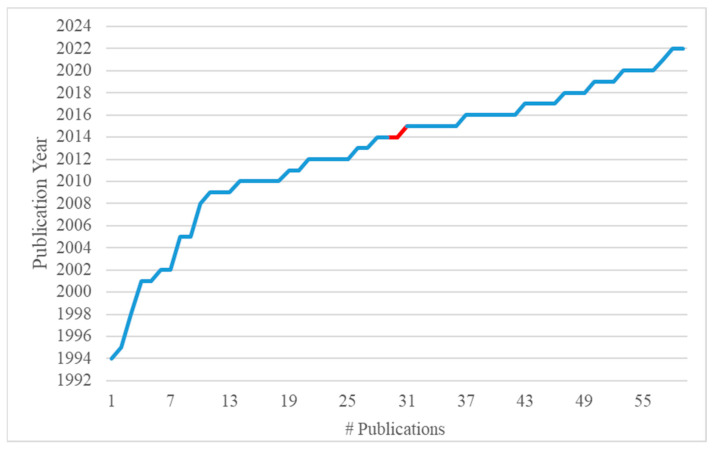
Annual rate of publications about FT-IR detection of EVOO adulteration over the period 1995–2022. Data retrieved from the Web of Science, Scopus and Google Scholar databases using various combinations of the keywords “virgin olive oil” AND “adulteration” AND “FTIR” in the search topics (last search: July 2022). The red segment depicts the shift to more systematic research during the last quarter of the publication period (2015 onwards).

Given the inclusion criteria, 32 journal articles that covered the entire publication period (27 years), were considered for further analysis in this study. Figure 2 shows the changes in the annual rate of publication of relevant papers since the early 1990s. It is clearly illustrated that the rate increased almost exponentially after the pioneering works of Wilson and colleagues [47,48,49]. It is also striking that almost 40% of the papers were published from 2015 onwards, which verifies that research on the particular topic has become more systematic in the last decade (Figure 2).

### 3.1. Field of Application

**Figure 3:**
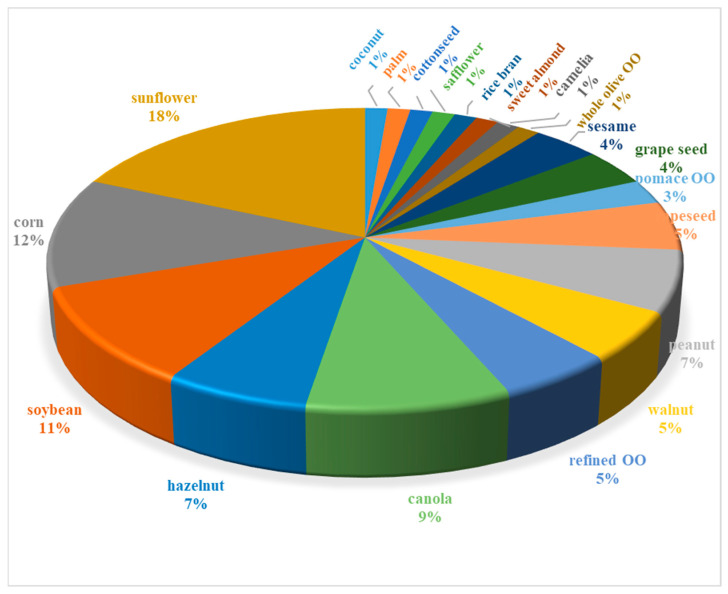
Frequency of records about the edible oils that were chosen to represent different botanical origin or process-type adulterants of EVOO in the articles under review.

Almost all of the eligible studies focused on discriminating mixtures of Extra Virgin Olive Oil (EVOO) with cheaper refined oils of various botanical origin (from vegetable, seeds and nuts) from pure EVOO. Mixtures with lower grade olive oil (refined olive oil (OO), pomace OO, OO from whole olive fruits, etc.) as process-type adulterants have also been investigated (Figure 3).

Refined sunflower, corn and soybean oils were, by far, the three most frequently investigated types of EVOO adulterants representing real cases of cheap, readily available substitutes in various local markets of the world. As they are typically devoid of chlorophyl or carotenoid pigments and much richer in polyunsaturated FAs than virgin olive oil, their FTIR spectral characteristics are easily discriminated from those of the authentic product. Thus, the probability of detection in binary EVOO mixtures via the proposed FTIR-based methods is expected to be high. In contrast, detection of blends with refined oils that are high-in-oleic acid is quite more challenging. It seems that process-type adulterants, such as refined olive and olive pomace oils, but also refined hazelnut oil, were investigated mostly in earlier studies published before 2015 [27,47,49,50,51,52,53]. Other botanical origin-type adulterants that are rich in oleic acid (e.g., from canola seeds and peanut) have gained popularity in studies published after 2015 [54,55,56,57,58,59,60]. At the moment, it cannot be estimated whether the shortfall in global edible oil supplies due to current geopolitical circumstances (e.g., Ukraine crisis) and the high increase in domestic market prices may prompt new types of fraud in the olive oil sector. Those conditions urge the need for implementation of efficient non-destructive screening tools and early warning signals in the official controls.

### 3.2. Sampling Methodology and Reference Samples

**Figure 4:**
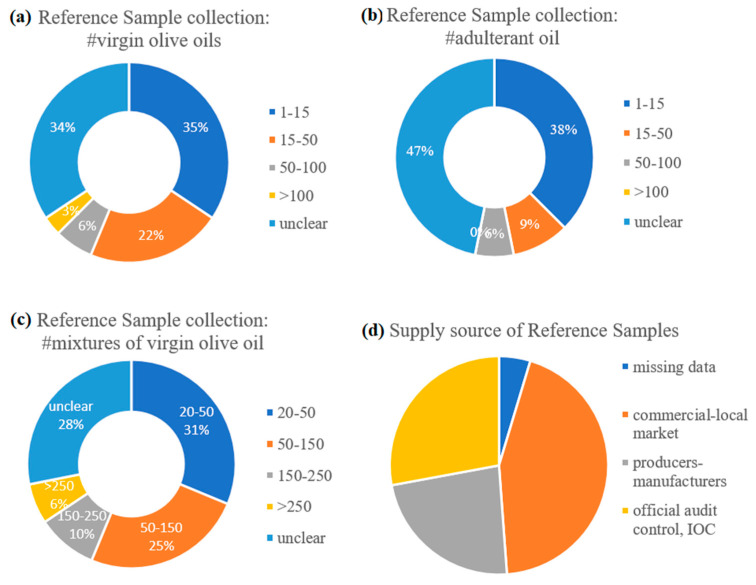
Recorded data about the size of reference sample collections for (**a**) virgin olive oil, (**b**) pure adulterant oils and (**c**) mixtures, along with (**d**) their supply sources in the EVOO adulteration studies.

The schemes in Figure 4a–c illustrate methodological trends regarding the size and the supply source of the sample collection used to create the reference spectra in each study. It is striking that almost 1/3 of the relevant publications reported poor details about their sampling method. The rest of the studies were classified basically into two groups that both corresponded to relatively small collections of reference spectra. The latter corresponded to either less than 15 or from 20 to 50 different samples of pure EVOO. In cases where the collection contained only a few average spectra (up to 15), they would rather represent different commercial EVOO brands with no tracking records on origin and production year. Very often, different batches of the same brand were purchased from the local market and mixed before analyses. Other investigators used mixtures of different commercial brands to reduce the heterogeneity of the reference sample set. Thus, compositional variation of authentic EVOO due to, e.g., cultivar, origin of production or harvest year, was seldom provisioned in the reference spectral data of the adulteration studies under review. When this condition was met, samples representing different geographical origin would be purchased from various international markets [27] or from producers [61] without any complementary purity control. In the cases where the collection counted up to 50 [49,50,53,55,62,63,64] and even more than 90 average spectra for EVOO [59,65], the sampling pattern was more complex; it seems that multiple sources other than the local market were used for the collection of pure EVOOs. Most probably, the researchers collaborated with producers, manufacturers, other colleagues, national authorities and official laboratories, as well as the IOC, to gain confidence about cultivar/geographical origin or quality parameters of the collected oils. The use of an in-house reference spectral library with available metadata for authentic EVOO/VOOs has also been exploited, as reported very recently [59]. Our survey showed that regardless of the supply source, only a third of the studies under review have attained accompanying analytical data with, e.g., fatty acid content of the samples, as additional purity indices of their reference samples [50,58,59,64,65,66,67,68,69,70,71].

As far as it concerns the sampling of refined vegetable or seed oils used as EVOO adulterants, details are poorly recorded (Figure 4b); the available data signify that collections per type of botanical origin are rather small (e.g., 1 up to 10 samples) and may represent a possible batch-to-batch compositional variation.

When it comes to the oil blends used as reference materials in adulteration studies, the dominant approach is to prepare the mixtures in-house with incremental dilution of the base EVOO, i.e., by simply mixing various volumes of a base EVOO with adulterant oils. The issue of poor representativeness may also refer to those blends. For example, each mixture component usually corresponds to a blend or individual sample that is selected arbitrarily from the whole collection to represent a given botanical origin. Details about the rationale behind the sampling and mixture design are often missing or not underlined in the published papers; thus, further assumptions cannot be made. In the majority of the studies, binary mixtures of authentic EVOO and a single adulterant oil were produced over widely varying volume ratios useful for calibration purposes. In such cases, the working levels could be much lower or higher than those that may stimulate fraud for economic profit.

Ternary or more complex mixtures were rarely studied using non-destructive protocols for FTIR spectral acquisition [58,59,63,69,70,72]. Table 1 summarizes the recorded details about the composition of the complex mixtures. The data show that the mixture preparation part is often poorly or not accurately explained. For example, Rohman and collaborators reported that “the composition of EVOO and others in their ternary and quaternary mixtures was randomly designed in order to avoid the correlated concentration profiles” and cited another paper for deeper detail about their design [73]. Three out of the total studies dealing with complex mixtures focused on investigating blends that contain >60–70% EVOO [58,59,63], and they pose a much greater challenge for attaining highly sensitive FTIR-based predictive models. It is worth noting that the most recently published ones explored ternary mixtures that bear a close resemblance to the authentic product with regard to their fatty acid profile and contents. The preparation protocols simulated different practices of fraud, e.g., dilution of the base EVOO with different volumes of a mixture of adulterant oils that has a standard composition (refined rapeseed and peanut oils, 1:1 *v/v*) [58] or with a fixed volume of an oil mixture that varies in relevant composition (refined canola, hazelnut or safflower oils, at interchanging volume ratios) [59] (see Table 1).

### 3.3. Spectra Acquisition Conditions

**Figure 5:**
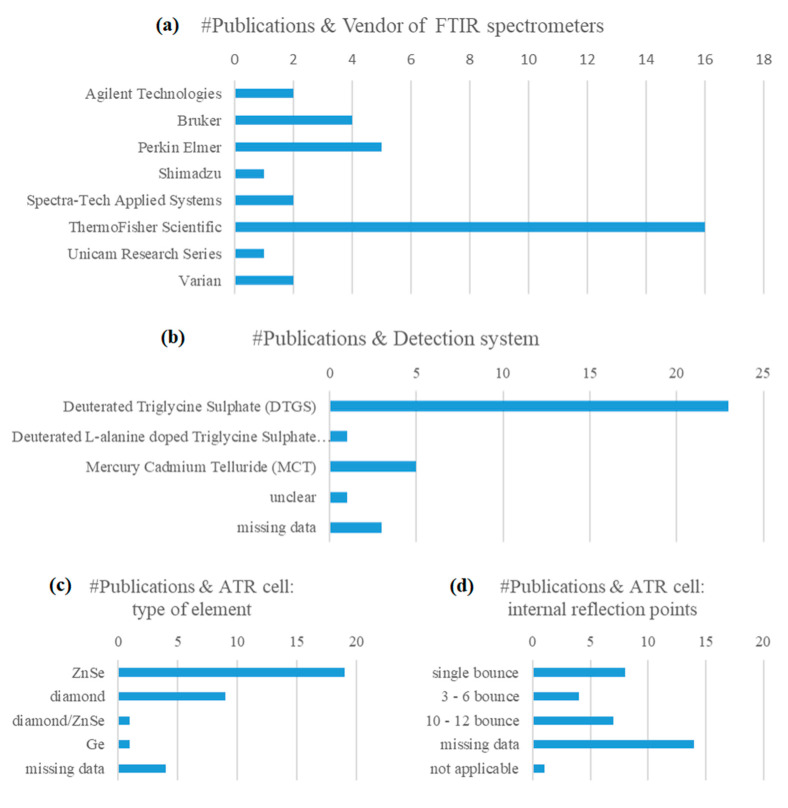
Data about the FTIR spectra acquisition conditions: (**a**) instrument models; (**b**) detectors; (**c**) ATR cell element types and (**d**) reflection points that were employed in the literature studies under review.

A standardized operating procedure for the preparation of the sample and subsequent measurements is necessary to evaluate data with the same statistical methods and develop sustainable models [74]. Our literature search indicated that the instrumental parameters and conditions of measurement are presented with highly varying degrees of detail in the articles under review. Reasonably, a variety of instrument models and sampling accessories from different vendors, as well as measurement protocols, have been used over the years. Relevant trends are highlighted in Figure 5a–d. Deuterated Triglycine Sulphate (DTGS) coupled with KBr optic system is a common detector system of FTIR spectrometers and, by far, the most frequently reported in the articles under review. Mercury-cadmium-telluride (HgCdTe, MCT), being more sensitive than common DTGS detector, but with lower dynamic range, plus the requirement for cooling (e.g., liquid nitrogen), followed next in frequency of report [27,50,53,58,62].

Based on the recorded data, the publications could be broadly clustered into separate groups according to the model and ATR cell characteristics of the FTIR instrument that was used in the study. Publications that were authored by the same group of researchers were excluded at this stage. Next, articles from different groups were pinpointed as of interest for coupling and comparison (e.g., [50] vs. [53], [54] vs. [65], [62] vs. [75], [67] vs. [76]) [50,53,54,62,65,67,75,76]. Only two shared a common objective, that was, detection of adulteration with sunflower oil. Apart from the detector (MCT vs DTGS), the sample measurement protocol was completely different. For example, it involved either deposition of the oil sample into an ATR cell holder and scanning the entire mid-infrared region [75] or dipping a fiber-optic probe with a diamond ATR sensor into the oil under study and scanning over 3000–600 cm^−1^ [62]. Despite the variation in analytical instrumentation and sample acquisition conditions, both studies claimed very small prediction errors (<3%) of their FTIR-based quantitative models for detecting sunflower oil in EVOO.

In almost half of the studies, the ATR cell accessory consisted of a horizontal plate equipped with a crystal window made from ZnSe and multiple internal reflection geometry. Alternatively, it could bear a single or 3-reflection point diamond cell. The retrieved data show that the cell accessory would be temperature-controlled in very few cases, e.g., [57,65]. As a general trend, the FTIR spectrum was acquired after removal of the background noise against the empty ATR cell (air), with a resolution of 4 cm^−1^ and by accumulating 32 interferograms. Most frequently, the samples were scanned over the entire mid-infrared region that expanded from 4000 cm^−1^ until 650 ± 50 cm^−1^. The long wavelength cut-off limit would vary widely from 400 to 900 cm^−1^, regardless of the type of cell crystal used, e.g., [76].

Notably, more than half of the publications under review do not report any information about the temperature of oil samples during measurements, the amount of the test sample in the cell or the number of replicate spectra per sample. In the remaining studies (almost a third of the articles), it is inferred that most often, three replicate spectra were taken at room temperature using a “small drop” of oil sample, which would vary from a few μL to few mL, depending on the sampling accessory. It has to be stressed that the choice of sample temperature during the measurements could be critical for the repeatability and overall quality of the FTIR spectra, given that some of the potential adulterant oils (e.g., palm oil) are solid at room temperature. The issue was raised early enough by Wilson and colleagues [49], but thus far, it has been appraised only once, in a non-targeted detection approach [65]. Elaborate washing and drying of the ATR cell window is absolutely needed to avoid the “memory effect” of the oil sample on the surface of the crystal [52]. Details about this procedure were missing in almost 40% of the studies under review or described satisfactorily in the rest, especially those using a horizontal ATR plate for spectral measurements.

The use of hand-held, portable FTIR instruments was also reported very recently. In 2018, Pan and colleagues [55] compared the performance of hand-held versus benchtop FTIR spectrometers in the detection of EVOO mixtures with corn, sunflower, soybean, canola and peanut oils (5–45%, *v/v*). According to their results, the hand-held ATR-FTIR method could accurately recognize mixtures with 5% to 10% of common adulterant oils, despite spectral interferences due to water vapor or lower signal-to-noise ratio and lower sensitivity compared with the benchtop instrument. Two years later, Aykas and colleagues [65] used portable FTIR instrumentation that allowed them to scan a quite large sample set of reference samples consisting of pure EVOO or blended with VOO, refined olive oils and vegetable oils that were attained through an official control laboratory or the IOC. Their models were found to be 100% efficient in recognizing blends of EVOO with refined olive oil and corn, sunflower, soybean or canola oil in a non-targeted approach. They also found that quantification of major fatty acids, pyropheophytin and total phenol content through the FTIR spectral data was sufficiently accurate (standard error of prediction < 1.5%). The findings thus far signify that the potential of portable FTIR technology for on-field applications to the anti-fraud control of olive oil needs further research to assess its technical and cost feasibility.

### 3.4. Spectral Pre-Processing Schemes

**Figure 6:**
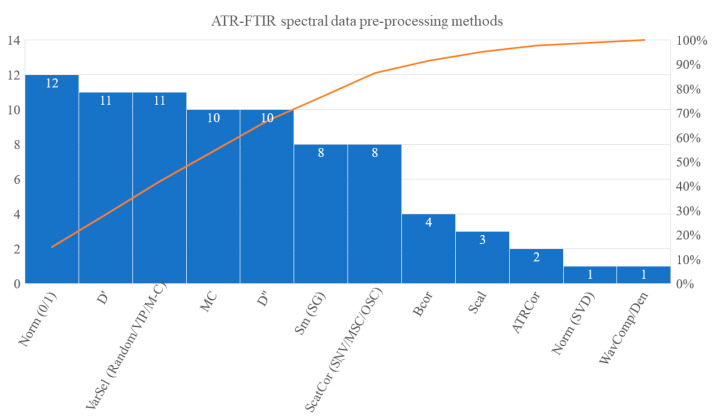
Pareto chart for the FTIR spectral data pre-processing methods that were most frequently reported in the EVOO adulteration studies under review. ATRCor, ATR correction; BCor, baseline correction; D’ and D’’, 1^st^ and 2^nd^ order derivatization; MC, mean centering; Norm, normalization according to 0/1 or Singular Value Decomposition (SVD); Scal, scaling; ScatCor, scatter correction via Standard Normal Variate (SNV), Multiplicative Signal Correction (MSC) or Orthogonal Signal Correction (OSC); Sm, smoothing via Savitzky–Golay (SG) method; VarSel, variable selection via random, Variable Important for Projection (VIP) or Monte-Carlo (M-C) algorithms; WavComp/Den, wavelet decomposition and denoising.

Variation in the spectral acquisition conditions, instrumental accuracy, inherent physical and compositional characteristics of the samples or experimental errors affect the quality of the obtained spectra and account for possible artifacts, such as noise, baseline shifts, slope, scatter effects, etc. Artifacts are not always easy to identify visually, as they involve combinations rather than individual bands. Thus, it is necessary to pre-process the raw data via various mathematical functions in order to remove these artifacts and obtain cleaned data that will better fit to the goal of chemometric analysis [41]. It is generally accepted that the method(s) of pre-processing can also introduce unwanted, irrelevant variation to the data; hence, their choice can be critical for the success of the entire experiment. However, objective criteria for the selection of the “best” spectral pre-processing methods for different types of spectral signals are not clear yet. In their review article, Lee et al. [43] stressed that it is not possible to draw any objective criteria, nor to propose a user-friendly tool to rapidly evaluate the performance of the pre-processing methods. In this section, we tried to deploy a strategy of selection and application of those methods by overviewing the relevant records in the studies under review. One out of the originally selected 31 publications [77] was excluded, as it did not apply chemometrics. The Pareto chart in Figure 6 shows the most widespread data-preprocessing methods, regardless of the sampling, measurement conditions or modeling methods used.

Simple normalization, 1st and/or 2nd order derivatization, wavelength selection and mean centering were the most popular mathematical treatments applied to the FTIR spectra of EVOO and its blends before chemometrics. In almost a third of the reviewed publications, the selection of limited spectral regions was the primary step of the workflow. Half of those studies referred to visual inspection and removal of the low S/N regions above 3100 cm^−1^ and between 1800 and 2600 cm^−1^ as a means to improve sensitivity. Several others referred to post-processing evaluation of the chemometric results on data sets that corresponded to more selective spectral regions. However, the methodology of variable selection was scarcely discussed in these publications. Specific feature extraction algorithms included moving window, stepwise Partial Least Squares (PLS) weights and Variable Importance Projection parameter (VIP), interval- and synergi-interval-PLS (i-PLS, si-PLS, respectively) and the Monte Carlo Uninformative Variable Elimination (MC-UVE) [44], but also “automatic” selection and visual inspection of the spectra have been reported, e.g., [51,56,58,60,62,64,65,67,68,75,78]. The use of the Successive Projection Algorithm (SPA) that is more popular in hyperspectral data pre-processing has been recorded once in the course of an EVOO adulteration study that aimed at fusing FTIR and NIR spectroscopic data [66].

The observed methodology trends stem probably from the need to tackle one of the major difficulties encountered with FTIR spectroscopic analysis of EVOO mixtures, that is, the low selectivity of the signal output. In the spectra of the mixtures, the infrared absorption bands that could serve as markers of the compositional changes are extensively overlapped. This type of interference can be implicitly resolved by derivatization, mainly of the 2nd rather than of the 1st order. Derivatization may also remove baseline offset, although this type of interference does not usually appear in ATR-FTIR spectra of edible oils. However, derivatization, especially of higher order, may introduce noise; thus, it is better to use it after a simple treatment that removes noise, such as smoothing.

Smoothing via the Savitzky–Golay algorithm [33] or even using wavelets [38] were used occasionally in the EVOO adulteration studies under review. A similar trend was observed also for baseline correction. The publication history records imply that pre-processing by derivatization became more popular over recent years (after 2015), probably because the corresponding algorithms could be easily implemented through the commercial spectroscopy software that became available (e.g., TQAnalystTM, The Unscrambler, PLS Toolbox, XLSTAT). Normalization, which passes from an absolute to a relative scale, by dividing the intensity value at each wavenumbers of a single spectrum according to a pre-selected constant, such as the highest or total sum of intensity, the spectrum norm, etc., was referred to in almost a fourth of the publications. In most cases, range scaling making the maximum and minimum absorbance values equal to 1 and 0, respectively, was applied. Preference for this kind of pre-processing implies that a part of the spectral variation (e.g., intensity fluctuations) could be associated with inconsistencies of the test sample amounts in the ATR cell [43].

As data set pre-processing, i.e., applied column wise on the data matrix holding all samples, mean centering, which removes a constant offset, is usually recommended and applied. It should be emphasized that centering the columns of the data matrix is appropriate in a standard pre-processing procedure prior to chemometrics via projection techniques (exploratory analysis by Principal Component Analysis (PCA) [36,42] or regression analysis by PLS [37,39]. Only in this way is it possible to define PCs as the linear combinations of the (centred) variables [36,37,39,42,46]. In a few cases, scaling, e.g., autoscaling that sets each column to the same variance, has been applied [47,59,63]. However, autoscaling is usually not recommended, as it forces baseline contributions to count as much as peaks [41]. In general, all the particular pre-processing methods are selected because they facilitate subsequent application of projection methods and interpretability of the resulting scores plots [43,63]. For example, scattering correction with the Standard Normal Variate (SNV) algorithm that subtracts the mean and divides with the standard deviation was often combined with mean-centering to more effectively correct background and offsets in the EVOO spectra.

**Figure 7:**
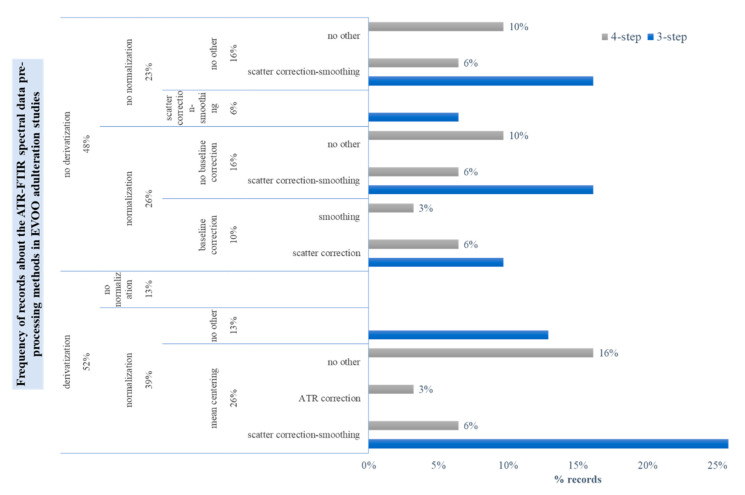
Outline of the most frequently used FTIR spectral pre-processing schemes in the EVOO adulteration studies under review.

Although PCA-based methods have been used quite frequently in the studies under review, almost half of the corresponding publications did not clearly record whether mean-centering was employed or not. In the great majority of the studies, the pre-processing scheme usually delimited multiple combinations of methods with no justification. Regardless of whether spectral variables were pre-selected or not, the spectra were pre-processed following one of the three generic workflows that are outlined in Figure 7. Thus, they would be either derivatized at some step of the pre-process or not derivatized at all. One way or another, normalization was often considered necessary, as commented above. Before derivatization, the spectra would get normalized [56,58,64,67,70,71], smoothed [59,65], mean-centered [51,72] or even baseline-corrected [78]. Less often, they would be derivatized first and then normalized, smoothed or mean-centered [27,54,69,76].

Comparison of each pre-processing method performance against unprocessed data is very rare in the relevant literature, e.g., [47,76,79]. Thus far, few research groups followed a post-processing optimization workflow to evaluate the fitting performance of those methods [27,56,59,64,67,68,71,72,79]. In particular, they rated quantitative, PLS or Principal Component Regression (PCR)-based calibration models of the corresponding data sets according to the root-mean-square error of cross-validation (RMSECV) and pinpointed the one with the lowest CV error as representative of the “best” pre-processing method(s). It is underlined that in some cases, the researchers made a decision after co-evaluating the prediction error (RMSEP) on independent test data sets, a strategy that is prone to data overfitting [64,72]. The chosen pre-processing scheme can be critical for the predictive performance of classification models, as well. In one case, the authors followed a rather misleading way to justify the selection of the “best” pre-processing method for the calibration data set based on the misclassification rate of the nearest centroid classification models on a given test data set in [76]. Apparently, the decision criteria can be multifaceted. In a recent article, it was reported that after scaling with the Pareto scaling method, the 2nd derivative, mean-centred FTIR spectral data for EVOO and complex mixtures fitted less efficiently to Partial Least Squares-Discriminant Analysis (PLS-DA) and one-class Soft Independent Modeling of Class Analogy (SIMCA) models [59]. Despite this, the sub-optimal models of the Pareto scaled data set were found more sensitive in detecting blends of similar FA composition to that of pure EVOO. It was suggested that discriminating information was probably hidden in the less intense spectral characteristics of the reference EVOOs that were revealed after scaling. The particular sub-optimal pre-processing method did not affect the detection of strong outliers in the reference data set. As pointed out by Engel et al. [41], it may be extremely difficult to pre-determine which combinations of pre-processing methods will correct for different spectral artifacts and highlight important information. Exploratory data analysis by PCA comparing scores and loadings plot before and after spectral preprocessing could help in understanding which effects are removed and/or if artifacts are introduced. Estimating the classification errors (in calibration phase) after choosing the best and worst pre-processing workflows with respect to model accuracy could help the decision-making. The uncertainty induced in this step can be minimized a priori if standardized experimental procedures up to the acquisition of the spectra are followed in order to remove sources of spectral interference and artifacts.

### 3.5. Exploratory Analyses

Exploratory approaches in the analysis of FTIR spectral data also became popular in recent years. In exploratory analysis, unsupervised approaches that attempt to identify the similarities and differences between samples by reducing the data dimensionality without any prior information about the data categories are highly appreciated [80]. Our literature survey showed that there were 10 relevant publications that reported the use of unsupervised techniques to reduce dimensionality of the FTIR spectral data [27,54,55,57,58,59,61,67,69,76]. Principal component analysis (PCA) was the chemometric technique of choice; some author groups [27,61,76] also employed Hierarchical Cluster Analysis (HCA), Continuous Locality Preserving Projections (CLPP) or Locally Linear Embedding (LLE) as alternative unsupervised methods for dimensionality reduction [61,81,82].

At this phase of model development, the original data matrix is decomposed to a new subspace with fewer dimensions, namely, the principal components (PCs), which are orthogonal and linear combinations of the original ones. PCs with high eigenvalues define the directions of the highest variability in the data, such that after the original observations are orthogonally projected onto the latent subspace, their exact location can be depicted through distinct score values. The most used algorithms to compute PCA are NIPALS (non-iterative partial least squares) or singular value decomposition (SVD) [36,42] that are encoded in the commercial spectroscopy software (e.g., TQAnalystTM, The Unscrambler, MATLAB-PLS Toolbox, XLSTAT). The employed algorithm is information not always easy to retrieve, and thus far, it was referred to only once [69]. A PCA model is fitted to a certain number of PCs, usually the percentage of explained variance guides the choice along with cross-validation (CV), and the goodness of fit is evaluated by computing R^2^ values. As few PCs should retain a high percentage of the original variance, their optimal number denotes the complexity of the model. The ultimate goal is to explore the data for similarities, meaningful trends and abnormalities or outliers. For example, in cases where FTIR is applied to monitor quality attributes during food processing, PCA score plots may grasp the dynamics of the process and explain the spectral data variance, which is of high value in routine process monitoring, as, e.g., in [25,83]. In the EVOO adulteration studies, PCA score plots were mainly exploited as a tool for visual assessment of possible separation among pure EVOO and adulterated samples. Exploratory PCA has been employed also as a strategy to detect outliers [59,69], an issue that seems to be ignored or not widely discussed thus far [49,58,62,65]. However, evaluation of outliers can be critical for the definition of the pure EVOO class boundaries, especially in the context of non-targeted spectroscopic analyses [84].

### 3.6. Modeling Strategies

The next step after data pre-processing involves different approaches, such as classification or calibration, depending on the aim of the study. The modeling step is always a two-phase procedure consisting of model development (calibration phase) and evaluation of model performance (optimization-validation phase). The strategy to evaluate the accuracy of the trained models involves, in turn, cross-validation (CV) or/and split-validation (SV) approaches. As a general rule, CV should serve the optimal setting of model meta parameters, such as the number of latent variables in PLS regression, while an external test set, not to be used at any point in the model calibration phase (neither to choose pre-processing, selecting spectral regions and so far), should be used to assess predictive performance (model validation). For the latter, the SV approach that involves a priori partition of the data into training and test sets is the most appropriate one, paying attention that calibration and test sets span a similar domain. In this way, the untrained data set is reserved for post-evaluation of the predictive model performance through a single set of accuracy statistics (e.g., standard error of prediction). Obviously, this validation step only covers an initial, most probably optimistic estimation of model performance, and future collected samples are really needed to assess the long-term model validity. In practice, the choice of the validation strategy could be also dictated by the size of the available reference sample collection or the goals of the study. For example, Leave One Out cross-validation (LOOCV) after sample order randomization was suggested as a compromise choice when resources to do reference measurement are limited [62].

Our survey pointed out several cases where clear information about the selected model validation schemes, i.e., the CV or SV methodology approach, the number and representativeness of samples in the split sub-sets could not be extracted from the recorded data [54,63,85]. Where available, the data showed that a calibration sub-set would most likely count for 60–90% of the total sample size. Few research groups [59,60,66,79] elaborated on the selection of a representative sampling algorithm (e.g., Kennard Stone and Onion methods) [40] that would help to retain even distributions of reference samples in the training and test sets, according, i.e., to compositional variation.

### 3.7. Calibration

Calibration models via PLS-R or PCR methods were developed in the majority of the EVOO adulteration studies under review. The authors used regression techniques exclusively or in combination with classification methods to make conclusions about the efficiency of the spectroscopic tool. In general, there were two different approaches of quantitative modeling, depending on the objective of the study. The first one aimed at evaluating the purity (%) of EVOO in the studied mixtures, regardless of the type of possibly co-existing foreign oil. In this case, the models were calibrated against the volume fraction of EVOO in the mixture (from 100% to 0%, *v/v*). In the second, more popular approach, the objective was to quantify a particular adulterant oil in the EVOO mixture. To increase the specificity of the methods, a variable selection pre-processing step would precede. Therefore, the X data matrix corresponded either to the whole FTIR spectrum [47,49,52,55,71,76] or to broad regions devoid of noise [27,50,57,59,61,63,69,72,79], or to shorter regions specific to the target adulterant(s) [51,56,58,60,62,64,65,67,68,75,78]. The methods used for this latter type of selection varied a lot, as discussed earlier.

#### 3.7.1. Case study I: Multivariate Regression of FTIR Spectral Data against Reference Fatty Acid Content Values

In the majority of the studies, the volume fraction of the target mixture component (EVOO or adulterant) spanning wide addition ranges over 0 to 100% of the total volume, constituting the y data. An alternative strategy considers a multivariate Y, including reference values from chemical compositional analyses, e.g., the % content in certain fatty acids. The latter may vary within the limits established by Commission Regulation (EEC) No 2568/91 [1,2] for EVOO’s major monounsaturated (oleic acid, C18:1; 55–83%), polyunsaturated (linoleic acid, C18:2; 3.5–21%) and saturated fatty acids (palmitic acid, C18:0; 7.5–20%).

The particular methodology of FTIR data calibration was reported in only two recently published articles [56,65]. It is worth noting that both exploited short spectral regions, and the second derivative spectral preprocessing, in order to obtain more robust and accurate calibration models for common EVOO adulterants (soybean, sunflower, corn and canola oils). Thus, variable selection in this phase of data modeling could be considered a switch towards a targeted analytical approach. For example, Filoda et al., using only a small collection of samples of unknown origin (10 pure EVOO and 5 seed oils), but known fatty acid composition, prepared 68 binary mixtures (4 types of seed oil x 17 levels of addition) and, finally, created a spectral data set with 91 observations, representing pure EVOO, pure refined seed oils (sunflower, soybean, corn and canola oils) and their mixtures. The iPLS and siPLS algorithms were used to screen which spectral regions where relevant, without a priori excluding small variance regions, which could also show significant fluctuations of major fatty acid content profiles (oleic, linoleic, linolenic acids) [56]. For instance, the variance over the carbon dioxide spectral region (2250–2330 cm^−1^), but also the region between 1860 and 1930 cm^−1^ (usually assigned to –CO–F) that are usually eliminated as irrelevant or noise, were revealed as important for the accuracy of their 10-factor FTIR-PLS-R models. The RMSEP values for linolenic (C18:3) and linoleic (C18:2) acids were found to be very low (<1 and 2%, respectively), much lower than that for oleic acid (C18:1) (<5%), and entailed a strong “proof of concept” about the potential of FTIR-based methods to recognize mixtures with oils that exceed the upper unsaturated FA content values of pure EVOO. The selected spectral features (3042−2727, 2331−2253, 1936−1858 and 1304−910 cm^−1^) were thus considered robust markers of atypical chemical composition. Following another methodology, Aykas et al. used a quite larger sample collection (>90 pure EVOOs and 70 mixtures with various types of vegetable oils) with accompanying metadata about the origin and purity, but also reference values for various quality indices (free fatty acids (FFA), peroxide value (PV), pyropheophytin (PPP), total polar compounds (TPC) other than the content in major fatty acids (palmitic, stearic, oleic, linoleic, and linolenic). In the variable selection step, they first eliminated irrelevant, noisy and unreliable variables from the FTIR spectra and then selected wavenumbers specific to the type of target molecule/quality index. Although details about the variable selection methodology are missing, it can be observed that the FTIR-PLS-R models of highest predictive ability (standard error of prediction < 1.0%) were those calibrated independently against FFA, TPC and PV values, along with palmitic, stearic and linolenic acid contents [65].

Overall, the proposed strategy of FTIR spectral data calibration requires large chemoinformatic database resources to build robust quantitative models. However, such models can offer more versatile solutions to the VOO anti-fraud control, as gross adulteration cases usually entail a high volume proportion of different types of foreign seed and vegetable oils that may alter the whole chemical compositional profile of the blend. Such fraudulent practices may thus distinctly leverage the total content in unsaturated or free FAs, suppress the content in polar phenolic compounds and cause relative content changes to several other constituents that are of diagnostic value in the mid-infrared spectral region.

#### 3.7.2. Case study II: Detection of EVOO Adulteration with Olive Pomace Oil

Thus far, two different research groups have proposed FTIR-PLS-R methods for the detection of olive pomace oil (OPO) in EVOO at concentration levels as low as 5% *v/v* [51,52]. This particular task is quite challenging because OPO represents a process-type adulterant of EVOO that resembles its chemical composition, especially regarding the profile of esterified FAs. Thus, certain compounds belonging to triterpene alcohols (e.g., uvaol, erythrodiol, aliphatic acids and waxes) that may mark the presence of OPO [86] are expected to contribute only with skeletal vibrations over the fingerprint, mid-infrared region, as shown in [52]. Table 2 presents data about the accuracy statistics and performance of the published methods. Overall, clear differences in the experimental design and strategy for data analysis were evidenced. As a result, the model that was trained with 1st derivative, mean-centered spectral data from the fingerprint region and corresponded to replicate measurements of only 3 mixture combinations (ref no [51]) was found less complex (4 vs. 11 Latent Variables), but also less accurate (REP = 16.4 vs. 3.3%), than the one that had been trained with multiplicative scatter-corrected spectral data from the whole mid-infrared region for 21 mixture combinations. External validation was performed with untrained data from either two new mixture combinations of the same oils [52] or new replicate spectra of the same combinations of the same oils used for calibration [51]. The recorded data indicate different degrees of model overfitting in the course of training and development, which probably account for the evidenced difference in the REP values of the two models. These results also highlight the need for representative sampling in both training and test sub-sets in order to optimize the predictive performance of the quantitative FTIR-based models.

### 3.8. Discirminant Classification

Classification models were also very popular among investigators in the field, as they were proposed in almost 2/3 of publications under review. In certain studies, the FTIR spectral data had been analysed only with classification methods [49,53,54,58,59,60,79]. Linear Discriminant Analysis (LDA) or Partial Least Squares Projection to Latent Structures-Discriminant Analysis (PLS-DA) were, by far, the most frequently used algorithms. In PLS-DA, the focus matrix, **Y**, expresses the class membership of the training set using one 0/1 dummy variable per class [45]. One of the most used classification rules consists of assigning a new sample to a certain predefined class when the predicted **y**-value for that class is higher than a threshold (e.g., 0.5) [45]. In the discriminant approach, each sample is always assigned to one of the classes, even if it does not belong to any of them. Other classification methods, such as the Nearest Centroid, which computes a centroid (mean) for each class and compares the FTIR spectra of a new sample to each of the class centroids, or the k-Nearest Neighbor (kNN), were also reported [61,76]. Neural networks (NN) and Support Vector Machines (SVM), which are popular in the machine learning community, have been scarcely exploited in the EVOO adulteration studies under review, e.g., [49,58,61].

### 3.9. Class Modelling Methods in Non-Targeted Approach

Over the last five years, class modelling methods, such as the Soft Independent Modeling of Class Analogy (SIMCA) [34,44], that are more appropriate to solve “asymmetric” classification problems, such as food fraud detection [87], became more popular in the field of EVOO authentication. SIMCA helps to identify systematic variation of samples that belong to a single class and then define its boundaries via different distance-based rules that resemble outlier detection techniques [44]. The probability of an unknown sample belonging to each class is modelled independently. According to Gao et al. [6], the use of one-class instead of multiclass classification is more appropriate because it can simplify the criteria into the typical/atypical question and help to make a decision of whether the product conforms or not to purity specifications. In contrast, two- and multiclass classification is quite more challenging, not only in determining relevant thresholds for each class, but also in acquiring truly representative sample sets as reference for classifications. SIMCA, as any class-modelling technique, differs from other supervised techniques because it does not force class assignments if the distance to the class model of an unknown sample exceeds the upper limit for every modelled class. However, it could be sensitive to undersampled data for the modelled category because the class boundary can be not well defined in those cases. Thus, a sample may not be assigned to a class either because it is an outlier (correctly) or because it comes from a class that is not sufficiently well represented in the model (incorrectly rejected in this case) [65].

Our literature survey showed that thus far, both one- and multiclass approaches have been applied to EVOO adulteration studies, resulting either in binary class models (authentic EVOO vs. EVOO mixtures), e.g., [59,63,65] or/and in multiple ones (e.g., authentic EVOO vs various types of mixtures depending on their composition), e.g., [55,61,65]. In any case, a well-defined target class that represents authentic EVOO samples was necessary to train those models. In an alternative approach, Georgouli and co-workers [61], using few samples of authentic EVOO (Italian and Greek origin), created a reference set of 256 binary mixtures with hazelnut oil. These mixtures represented different combinations of the four base oils with four samples of crude and refined hazelnut oil at varying volume ratios from 0 to 100%. Then, multiclass SIMCA models (among other methods), referring to concentration ranges of low or high resolution (4 vs 10 classes, respectively), were assessed, indicating better classification rates in the former case.

Special emphasis will be given to the records about one-class modeling methods in EVOO adulteration studies, as they are suggested for non-targeted authenticity testing [15]. In this approach, only the class of authentic samples is modelled; this is because in practice, the falsified samples do not really constitute a class, but an amorphous collection of adulterated, diluted, spiked, etc. samples that do not share common characteristics [87]. In line with the non-targeted approach, Gurdeniz and Ozen were the first to propose a one-class FTIR-SIMCA model for detecting EVOO adulteration. Their model was found efficient in recognising mixtures with corn and sunflower oils at levels > 5% *v/v*. Based on visual observations, the authors suggested that the discriminating power of their model was due to spectral variance at around 3000–3010 and 2923 cm^−1^, at 1377 and 913–914 cm^−1^ that can be diagnostic of changes in the unsaturation level, the relative content of monounsaturated fatty acids, along with that of triolein, respectively. The non-targeted approach was reported again many years later, in the article published by Aykas and colleagues [65]. Their one-class SIMCA model was found 100% efficient in recognising counterfeit EVOO products (that were known to contain corn, sunflower, soybean and canola oil at unknown levels) and 89% efficient in identifying blends with lower grade olive oil (mixture of VOO and OO). It is emphasized that a portable instrument was used to scan pure EVOO and different types of blends with widely varying composition in major fatty acids. In this case, the discriminating power was attributed to bands at 2830–2930, 1777, 1705, 1672, 1412 and 1377, as well as 1107 to 1172 cm^−1^ (see Figure 1 for peak assignment). Very recently, another one-class FTIR-SIMCA method that investigates adulteration of EVOO with blends of adulterants that simulate the composition of the authentic product was developed and proposed by some of us [59]. In that study, a reference spectral library for pure EVOO and VOOs, accompanied by metadata for harvest year, geographical origin, cultivar and/or fatty acid composition and other quality indices, was exploited. Six base oils of different geographical origin and varying content in some quality attributes (e.g., TPC), along with different samples of refined canola, hazelnut and/or safflower oils, and known fatty acid composition were used to prepare 101 binary and ternary mixtures of EVOO with adulterant oils at a standard addition level (20% *v/v*). The one-class SIMCA model was trained with 2^nd^ derivative FTIR spectral data for samples of 47 authentic EVOOs that covered different harvest years, cultivars and geographical origins against those of 73 mixtures. Validation was performed by creating several external sets of EVOOs from different harvest year, cultivar and origin, lower grade olive oils (VOO) or different combinations with adulterant oils, signifying high overall predictive power, >92%.

Based on the recorded data, the aforementioned one-class FTIR-SIMCA models detected challenging cases of adulteration, i.e., blends with lower grade olive oil [65] or with a mixture of 5% canola and 15% hazelnut oils [59] as atypical EVOOs, in line with the principle of non-targeted authenticity testing. The loading plots exposed that spectral variation at 2820–2860 and above 2920 cm^−1^ (especially around 3001–3005 cm^−1^) were the most discriminating features of the reference EVOO mixtures. The region between 950 and 976 cm^−1^ (isolated trans-double bonds) was found important for identifying mixtures with refined oils that are richer in trans-fatty acids.

To gain further insight into the identity or the concentration of the foreign oil(s) in the particular type of counterfeit products, one can consider combination with quantitative methods, i.e., PLS-R. In this context, a data fusion strategy that may combine the outputs of FTIR with those of a different non-destructive spectroscopic technique, such as NIR, has emerged as a way to increase the reliability of multivariate calibration methods as compared to using a single technique [88]. Similar efforts in the field of EVOO adulteration are on their way [66,89] and pave the step toward new, more advanced applications of the FTIR technique.

### 3.10. Method Performance Criteria

Almost every qualitative method proposed in the studies under review was evaluated in terms of model accuracy statistics (number of optimal factors, summary of fit-to-model), regardless of the algorithm used for multivariate calibration. Correct classification rates (CCR), mahalanobis distance metrics in Coomans’ plots and Fisher statistics are also very popular tools for assessing the performance of those models. Other important figures of merit, such as the sensitivity (the percentage of samples that are correctly recognized as members of the target class) and specificity (the percentage of samples from other classes that are correctly assigned as suspect/non-compliant) of the predictive models, are currently gaining importance, especially in the research field of non-targeted food authentication. Standard guidelines about how to set the threshold class value (boundary between the two classes) that optimizes both the sensitivity and specificity of the predictive one-class models [90] or how to establish a regular method evaluation protocol in line with the principles of quality assurance [84] are yet to be addressed.

## 4. Concluding Remarks and Future Perspectives

In summary, our literature review and meta-analysis of the retrieved data verified that research beyond the “proof of concept” stage is needed to develop accurate, robust and fit-for-purpose FTIR-based methods for non-targeted detection of VOO adulteration. The exposed trends in the research methodology thus far showed that non-representative sampling, along with the uncontrolled conditions of oil sample treatment and spectra acquisition, are underestimated sources of systematic errors that need to be taken into account more carefully in the future. In some cases, misconduct in spectral data curation and multivariate analysis may stem from a lack of technical knowledge or overexploitation of mathematical and statistical treatments. All these different aspects of research methodology define the complexity of the analytical problem, which is related to non-targeted authenticity testing. In the future, data fusion strategies that aim to combine the signal outputs of different non-destructive spectroscopic techniques (i.e., FTIR with NIR, Raman and/or low-field NMR) are expected to increase the reliability of multivariate calibration methods, as compared to those based on a single fingerprinting technique. New approaches in data analysis, along with tendencies for integration with novel optic systems, mid-infrared hyperspectral sensors and miniaturization of spectrometers, will most probably boost the need to standardize the FTIR experimental protocols. Standardization is essential to produce a reproducible molecular fingerprint of virgin olive oil, without sophisticated pre-processing before statistical evaluation. Building comprehensive, chemoinformatic databases for exploratory analyses will certainly assist non-targeted screening. Above all, the development of FTIR predictive models for VOO adulteration seems to be a decision-making process that requires close collaboration among scientists from multidisciplinary fields and expertise.

## Figures and Tables

**Table 1:** Recorded details about the composition of complex mixtures of olive oil with adulterant oils in the EVOO adulteration studies under review (in chronological order).

Combination of Oils		Mixture Composition (%)		No. of Study under Review (Cited Reference)
	Preparation	A—Adulterant Oil (Oil 1:Oil 2)	B—EVOO (*n* of Samples)	
EVOO–CO–SuO	Incremental addition of A to B—varying composition of A	2–20 (0:1, 1:3, 1:2, 1:1, *v/v*)	80 (*n* = 5) 85 (*n* = 3) 90 (*n* = 3) 95 (*n* = 3) 98 (*n* = 3)	[63]
EVOO–GSO–RBO–WNO	Random design	0–100 (miscellaneous ratios)	0 (*n* = 4) 2.5–50 (*n* = 19) 50–98 (*n* = 3) 100 (*n* = 1)	[72]
OO–VCNO–PO	Random design	0–100 (miscellaneous ratios)	0 (*n* = 2) 2.5–40 (*n* = 9) 40–65 (*n* = 12) 100 (*n* = 1)	[70]
EVOO–SuO–RSO and EVOO–HOSuO–RSO and EVOO–HOSuO–SuO	Incremental addition of A to B	0–100 (miscellaneous ratios)	0 (*n* = 9) 10–40 (*n* = 34) 50–90 (*n* = 20) 100 (*n* = 1)	[69]
EVOO–PNO–RSO	Incremental addition of A to B—standard composition of A	~2–40 (1:1, *v/v*)	~60–98 (*n* = 120)	[58]
EVOO–CO–HO–SafO	Fixed volume addition of A to B—varying composition of A & G—optimal simplex design	20 (0:1, 1:7, 1:3, 1:1, 3:1, 7:1, 1:0, *v/v*) 13 (1:1, *v/v*) 10 (1:0, 0:1, *v/v*)	80 (*n* = 137) 87 (*n* = 3) 90 (*n* = 8) 100 (*n* = 85)	[59]

CO, corn oil; EVOO, extra virgin olive oil; GSO, grape seed oil; HO, hazelnut oil; HOSuO, High-oleic sunflower oil; OO, olive oil; PO, palm oil; PNO, peanut oil; RBO, rice bran oil; RSO, rapeseed oil; SafO, safflower oil; SO, soybean oil; SuO, sunflower oil; VCNO, virgin coconut oil; WNO, walnut oil.

**Table 2:** Methodology design for the FTIR-PLS-R detection of olive pomace oil in EVOO (data extracted from [51,52].

No.	Type of Adulterant Oil/Country	Reference Samples ^1^ (*n*)	OPO-VOO ^2^ (%, *v/v*)	C_working_ Levels	Working Spectral Region (cm^−1^)	Spectral Data Pre-Processing	Criteria for Selection of Optimal LVs ^3^	LVs (*n*)	n_cal_ /n_val_ Spectra	PRESS ^4^	Fitness to the Model (R^2^)	REP ^5^ (%)
[52]	Olive Pomace Oil/USA	4	5:95 to 95:5	21	4000–650	MSC	RMSECV ^6^	11	21 × 3/21 × 1 ^7^	0.122	0.991	3.3
[51]	Olive Pomace Oil/Italy	- ^8^	5:95 to 30:70	5	1876–912	MC, D’, MW (10p)	F ratio of PRESS Haaland & Thomas criterion, 1988	4	3 × 3/2 × 3	0.002	0.973	16.4

^1^ used for mixture preparation; ^2^ OPO, Olive Pomace Oil; VOO, Virgin Olive Oil; ^3^ LV, Latent Variables; ^4^ PRESS, Predicted Residual Error of Sum of Squares; ^5^ REP, Residual Error of Prediction; ^6^ RMSECV, Root Mean Square Error of Cross Validation; ^7^ replicate spectra; ^8^ denotes missing data.

## Data Availability

Not applicable.

## Supplementary Materials

The following are available online at https://www.mdpi.com/article/10.3390/molecules28010337/s1 (accessed on 1 January 2023), Glossary of terms and basic descriptions of chemometric methods referred to throughout the text.
