# Supplementary material for: Toward the Non-Targeted Detection of Adulterated Virgin Olive Oil with Edible Oils via FTIR Spectroscopy & Chemometrics: Research Methodology Trends, Gaps and Future Perspectives
Source: Molecules. 2023 Jan 1;28(1):337. doi: 10.3390/molecules28010337 (PMC9822006; doi:10.3390/molecules28010337)
Supplement: Supplementary file 1 [file molecules-28-00337-s001.zip › molecules-2034531-supplementary.pdf]

Review

Toward the Non-Targeted Detection of Adulterated Virgin Olive Oil with Edible Oils via FTIR Spectroscopy & Chemometrics: Research Methodology Trends, Gaps and Future Perspectives

Stella A. Ordoudi [1,] *, Lorenzo Strani [2] and Marina Cocchi [2]

1 Laboratory of Food Chemistry and Technology, School of Chemistry, Aristotle University of Thessaloniki (AUTh), GR-54214, Thessaloniki, Greece
2 Department of Chemical and Geological Sciences, University of Modena and Reggio Emilia (UNIMORE), Via Campi 103, 41125 Modena, Italy
* Correspondence: steord@chem.auth.gr

Supplementary material:

Glossary of terms and basic descriptions

Principal Component Analysis (PCA)

PCA is a multivariate tool for the detection and evaluation of similarities, differences, outliers and cluster tendency on experimental data. Through PCA it is possible to reduce the dimensionality of the data, as it transofrms the original variables in few new, uncorrelated variables, the Principal Components (PCs). The first PC is constructed by finding the direction of the n-dimensional space (n=number of variables) which incorporates the largest source of variability in the data. The second PC, orthogonal to the first, is constructed involving the largest variability not expressed by the first one, and so on for the subsequent PCs. In this way, only the first PCs contain the relevant information, whereas further PCs include only random noise. Equation S1 describes how a data matrix X composed by *m* rows and *n* columns is decomposed by PCA:

$$X = TP^T + E \tag{S1}$$

T is the scores matrix, that holds the information on how the samples are related to each other, whereas P is the loadings matrix, that express the influence of the original variables on the scores. E is the residual matrix. Hotelling T^2 and Q residuals parameters are used to evaluate a PCA model and to assess if a sample can be classified as an outlier. T^2 statistic represents the distance of a sample in the space of significant PCs (i.e. in the model space), whereas Q describes the distance of a sample from the model space, basically assessing if the variability of a sample can or cannot be explained efficiently.

Principal Component Regression (PCR)

PCR is a technique used for the multivariate linear regression that is based on PCA. First, it calculates a PCA decomposition of the data matrix X, the predictors matrix, and secondly it computes a multilinear regresion model on the obtained scores to predict Y, the response matrix. Since in PCR the data are represented in a reduced-dimensional variable space, it is critical to decide the proper number of PCs. In fact, models built with

too few components could not be able to fit X well and, as a consequence, predict Y accurately. On the other hand, choosing too many components can lead to overfitting X and Y, computing models with unstable and unreliable prediction performances.

Partial Least Squares (PLS) Regression

PLS is a method used to perform a linear multivariate regression, evaluating the correlation between two data matrices. Its main advantage is that deals efficiently with data matrices containing more variables than samples, especially if those variables co-vary. PLS performs a simultaneous decomposition of X and Y with the aim to explaining to the greatest extent the variability of X and finding the best correlation with Y. The algorithm calculates the decomposition of X and Y in the same way of PCA does (Equations S2 and S3):

$$X = TP^T + E \qquad \text{(S2)}$$

$$Y = UQ^T + R \qquad \text{(S3)}$$

The properties of both scores and loadings are the same described for PCA, with the exception that in PLS the components, called Latent Variables (LVs), are built explaining the variability in X that most influences the prediction of the responses in Y. Hence, as in PCR, the choice of LVs number is crucial to obtain reliable prediction models.

Partial Least Squares – Discriminant Analysis (PLS-DA)

PLS-DA is a multivariate classification technique belonging to the family of discriminant methods, which aim to identify the optimal boundaries among different classess while minimizing the classification error. It is based on PLS regression, meaning that exploit a low-dimensional space computed by maximizing the covariance with Y, but in this case the Y matrix, called dummy matrix, contains the information relatated to class membership for each sample. The classification is accomplished by choosing a proper threshold to the predicted Y values.

Figures of merits of regression models

Normally, to evaluate the performances of a regression model, two main parameters are inspected: R^2 and Root Mean Squared Error (RMSE). The latter can be expressed as Root Mean Squared Error in Cross-Validation (RMSECV) and Root Mean Squared Error of Prediction (RMSEP). RMSECV refers to the prediction error obtained performing the cross-validation on the X data matrix (internal validation), whereas the RMSEP indicate the prediction error obtained by projecting new samples on the prediction model (external validation). The R^2 is an index that measures the relation between the variability of the data and the correctness of the statistical model used.

Soft-Indipendent Modelling of Class Analogy (SIMCA)

SIMCA is a class-modelling multivariate classification method, which are based on finding similarities between elements from the same class rather than on the differencies among classes. In particular, SIMCA models each class indipendently by trying to capture the sistematic variability that characterize the samples of a certain class through PCA (more specifically through the calculation of Hotelling T^2 and Q residuals).

Hierarchical Cluster Analysis (HCA)

HCA is a clustering method that aims, as the name suggests, to build a hierarchy among samples (and among clusters of samples). As a first step, the two most similar samples are detected and the first cluster is formed. Starting from the second step, the user has to choose which will be the criteria to agglomerate the other samples/clusters (average linkage, centroid linkage, Ward's method etc.). The algorithm continues to form bigger clusters until a cluster containing all the samples is obtained.
